# Novel Oral Anticoagulant versus Warfarin in Cancer Patients with Atrial Fibrillation: An 8-Year Population-Based Cohort Study

**DOI:** 10.7150/jca.36468

**Published:** 2020-01-01

**Authors:** Victor Chien-Chia Wu, Chun-Li Wang, Yu-Tung Huang, Wen-Ching Lan, Michael Wu, Chang-Fu Kuo, Shao-Wei Chen, Pao-Hsien Chu, Ming-Shien Wen, Chi-Ching Kuo, Shang-Hung Chang

**Affiliations:** 1 Division of Cardiology, Chang Gung Memorial Hospital, Linkou Medical Center, Taoyuan City, Taiwan; 2 College of Medicine, Chang Gung University, Taoyuan City, Taiwan; 3 Center for Big Data Analytics and Statistics, Chang Gung Memorial Hospital, Linkou Medical Center, Taoyuan City, Taiwan; 4 Graduate Institute of Nursing, Chang Gung University of Science and Technology, Taoyuan City, Taiwan; 5 Divison of Cardiovascular Medicine, Arrhythmia Services Section, Rhode Island Hospital, Warren Alpert School of Medicine, Brown University, Providence, USA; 6 Division of Rheumatology, Allergy and Immunology, Department of Internal Medicine, Chang Gung Memorial Hospital, Linkou Medical Center, Taoyuan City, Taiwan; 7 Division of Rheumatology, Orthopaedics and Dermatology, School of Medicine, University of Nottingham, Nottingham, UK; 8 Department of Cardiothoracic and Vascular Surgery, Chang Gung Memorial Hospital, Linkou Medical Center, Taoyuan City, Taiwan; 9 Institute of Organic and Polymeric Materials, National Taipei University of Technology, Taipei, Taiwan.

**Keywords:** anticoagulation, atrial fibrillation, cancer, NOAC, warfarin

## Abstract

**Background:** Cancer patients with atrial fibrillation (AF) were excluded in the major clinical trials. We therefore investigated the efficacy and safety of novel oral anticoagulant (NOAC) versus warfarin in these patients.

**Methods:** Data were retrieved from Taiwan National Health Insurance Research Database during 2010-2017 for patients with AF, excluding those without cancer or >1 cancer, not using anticoagulant, switching of agents, patients age <18, and cancer and AF diagnosed >1 month apart. Primary outcomes are ischemic stroke (IS)/systemic embolism (SE), GI bleeding, major bleeding, intracranial hemorrhage (ICH), acute myocardial infarction (AMI), and death from any cause at 6 months and 1 year.

**Results:** After exclusion criteria and propensity score matching, there were 336 patients in each group. Patients on NOAC had significantly reduced IS/SE (HR=0.45, 95% CI=0.25-0.82), major bleeding (HR=0.21, 95% CI=0.05-0.96), and no ICH at 6 months. In addition, IS/SE (HR=0.42, 95% CI=0.24-0.74), major bleeding (HR=0.26, 95% CI=0.09-0.76), and no ICH at 1 year compared to patients on warfarin. There was no difference on GI bleeding, AMI, and death from any cause at 6 months and at 1 year.

**Conclusion:** In cancer patients with AF, NOAC were associated with significant reduced IS/SE, major bleeding, and ICH compared to warfarin.

## Introduction

Patients with atrial fibrillation (AF) and a calculated CHA_2_DS_2_-VASc score ≥1 in men and ≥2 in women requires anticoagulation therapy with warfarin or novel oral anticoagulant (NOAC) 1. Cancer is an important condition associated with development of AF 2,3. A higher cardiovascular risk factors has been noted in AF patients with cancer than without 4. In cancer patients, there is increased risk of venous thromboembolism due to cancer-related disturbance of homeostasis and hypercoagulable state 5. In addition, cancer patients undergoing chemotherapy frequently has complication of bleeding due to thrombocytopenia 6. In cancer patients with AF, currently there is no guideline to direct our clinical management and they are less likely to receive adequate treatment for the concomitant diseases. Traditionally, the major landmark trials of NOAC with dabigatran, rivaroxaban, apixaban, and edoxaban for use in AF excluded patients with cancer 7-10. Recently, edoxaban was found to be noninferior to subcutaneous low molecular weight heparin for the treatment of cancer-associated venous thromboembolism in the randomized clinical trial 11. However, there is still lack of evidence supporting the use of NOAC over warfarin in these cancer patients with AF. Therefore, the aim of this study is to determine the efficacy and safety associated with use of NOAC versus warfarin in these patients.

## Materials and Methods

### Data Source

Taiwan's National Health Institute Program started in 1995 and provides 99.5% coverage for the 23 million residents in Taiwan. The NHI Research Database (NHIRD) provides all dates of inpatient and outpatient services, diagnoses, emergency room visits, prescriptions, examinations, operations, and expenditures, and data are updated biannually. With over 95% of Taiwanese population consists of Han Chinese, our study is considered of uniform ethnic background. The Institutional Review Board of Chang Gung Memorial Hospital Linkou Branch approved this study (IRB No. 201800725B1).

### Study Patients

By searching electronic medical records from the NHIRD between January 1, 2010 and December 31, 2017, we retrieved patients with diagnosis of AF, using at least 1 inpatient or 2 outpatient claims for nonvalvular AF 12. Active cancer was defined as cancer receiving treatment or diagnosed within last 6 months from previous report 12 and by NICE guide in United Kingdom and a previously published report 13, therefore we did not enroll patients whose follow-up period >6 months. Patients with cancer were screened, with exclusion criteria of no cancer or more than one type of cancer, not using oral anticoagulant (OAC) of either warfarin, dabigatran, rivaroxaban, apixaban, or edoxaban, switching of OAC agents, and patient age <18. In addition we exclude patients with cancer and AF diagnoses >1 month apart and use of OAC and AF diagnosis > 1 month apart. Primary outcomes defined as ischemic stroke (IS)/systemic embolism (SE), GI bleeding, major bleeding, intracranial hemorrhage (ICH), acute myocardial infarction (AMI), and death from any cause at 6 months and 1 year.

### Study Outcomes and follow up

We selected efficacy outcome of IS/SE, AMI, and death from any cause, and safety outcomes of major bleeding, GI bleeding, and ICH at 6 months and 1 year. The major bleeding was defined according to principle or secondary diagnosis of hospitalization and emergency visit and any blood transfusion order, which included admission for any bleeding, required blood transfusion >2 U, and life-threatening bleeding or vital organ hemorrhage, which included ICH. Death from any cause was defined by withdrawal from the NHI program 14.

Disease was detected using International Classification of Diseases, 9th Revision, Clinical Modification (ICD-9-CM) and ICD-10 codes. Covariates included gender, age, medical history of diabetes mellitus, hypertension, hyperlipidemia, heart failure, renal insufficiency, peptic ulcer disease, abnormal liver function, peripheral arterial disease, prior ischemic stroke or systemic thromboembolism, old myocardial infarction, bleeding history, alcohol history, medication, CHA_2_DS_2_-VASc score, and HAS-BLED score. The comorbidity was defined as having two outpatient diagnoses or one inpatient diagnosis in the previous year. Similarly, usage of medication was retrieved based on claim data within six month before and after the index date.

### Ascertainment of Diseases and Comorbidities

Our study retrieved data from NHIRD, using ICD-9-CM and ICD-10 disease coding with diagnoses defined as discharge diagnosis or at least two consecutive clinic visits. The accuracy of the diagnosis of has been confirmed and validated in previous studies 15-17. In addition, Catastrophic Illnesses Registry, which all confirmed cancer patients are required to be registered, enhances the accuracy and completeness of data retrieval of patients with malignancy. The positive predictive value (PPV) of all cancers are 93.64% and the selected cancers are: cervical cancer, PPV 81.73%; colorectal cancer, 94.38%; esophageal cancer, PPV 94.45%; female breast cancer, PPV 91.86%; gastric cancer 91.84%; liver and intrahepatic ducts cancer, PPV 93.03%; lung cancer, PPV 94.90%; oral cavity, oropharyngeal, and hypopharyngeal cancer, PPV 94.40%; prostate cancer, 93.29%; pancreatic cancer, PPV 90.39% 16. In addition, the PPV of selected comorbidities are: diabetes, PPV 92%; hypertension, PPV 88.5%; hyperlipidemia, PPV 89.5%; heart failure, PPV 97.6%; myocardial infarction, PPV 92.0%; ischemic stroke, PPV 97.9% 17.

### Statistical Analysis

Each patient who was in warfarin group was matched to a patient in the NOAC group. We compared the baseline characteristics, comorbidities, and medication, CHA_2_DS_2_-VASc score, and HAS-BLED score between the study groups using t-test for continuous variable or chi-square test for categorical variable. We compared the risk of death from any cause between groups using a Cox proportional hazard model. We generated the plot of cumulative probability using subdistribution hazard function for time to event outcomes. A *P* value < 0.05 was considered to be statistically significant. No adjustment of multiple testing (multiplicity) was made in this study. All statistical analyses were performed using commercial software (SAS 9.4, SAS Institute, Cary, NC).

### Sensitivity Analysis

In order to validate our study findings and check for potential selection biases, we performed the sensitivity analysis. In contrast to the main analysis which we exclude patients with AF and cancer diagnosed >1 month apart, we re-analyzed the data with AF and cancer diagnosis >1 year, which is similar to ARISTOTLE study on apixaban that enrolled patients with cancer within the previous 12 months. The result of the main study was compared to different enrollment criteria to assess whether the primary findings would be modified.

## Results

### Study Population

**Figure 1:**
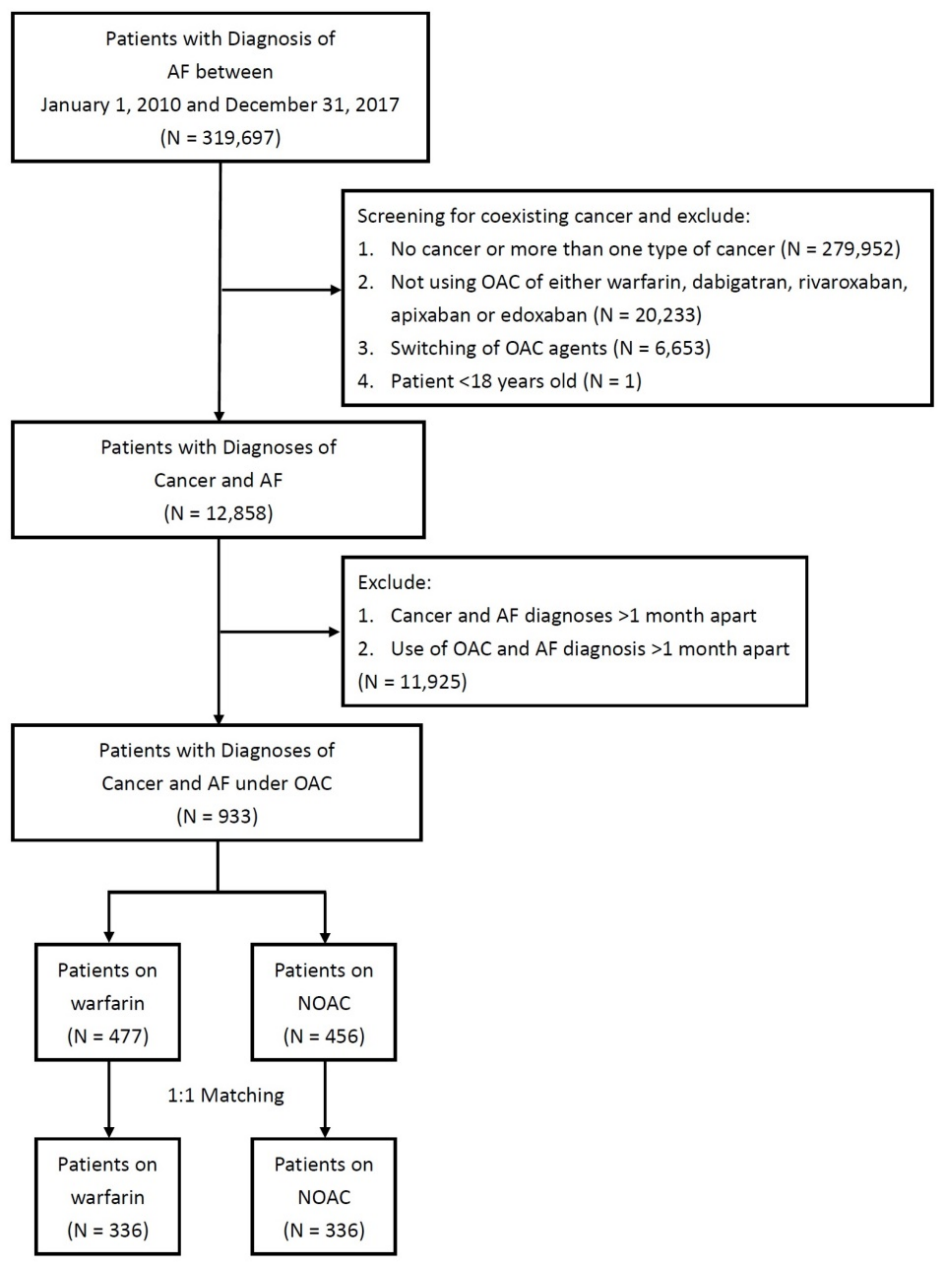
Study design and screening criteria flow chart for the inclusion of cancer patients with atrial fibrillation (AF). OAC, oral anticoagulant

There were 319,697 patients with a principal diagnosis of AF during 2010 and 2017 identified in the NHIRD. After exclusion criteria, there remained 933 patients with 477 on warfarin and 456 on NOAC eligible for analysis (**Figure 1**). After propensity score matching, there were 336 patients on warfarin and 336 patients on NOAC for study (**Table 1**). Before propensity score matching, patients who were on warfarin were younger, had lower prevalence of diabetes mellitus, hypertension, hyperlipidemia, abnormal liver function, peripheral artery disease, and alcoholic history. After propensity score matching there were no difference between the two groups. Before propensity score matching, CHA2DS-VASc score were 3.92±1.93 in patients on warfarin, and 4.34±1.97 in patients on NOAC. After propensity score matching, CHA2DS-VASc score were 4.20±1.89 in patients on warfarin, and 4.21±2.00 in patients on NOAC. Before propensity score matching, HAS-BLED score were 2.93±1.58 in patients on warfarin, and 3.38±1.42 in patients on NOAC. After propensity score matching, HAS-BLED score were 3.19±1.56 in patients on warfarin, and 3.25±1.44 in patients on NOAC.

### Patients on warfarin versus patients on NOAC

**Figure 2:**
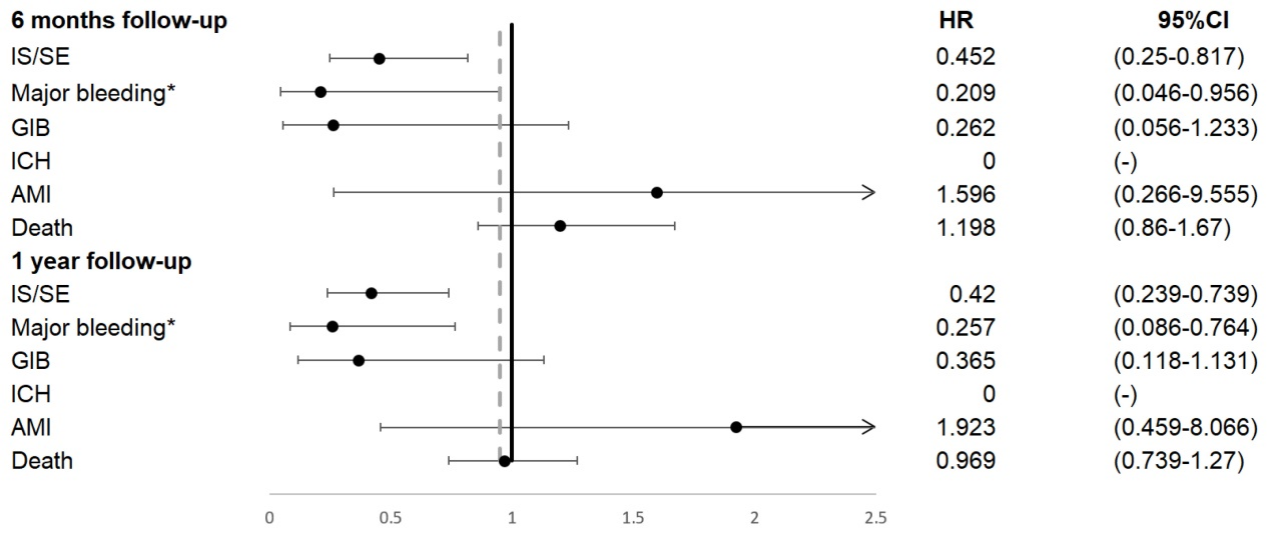
Primary outcomes occurred during 6 months and 1 year follow up. AMI, acute myocardial infarction; GIB, gastrointestinal bleeding; ICH, intracranial hemorrhage; IS/SE, ischemic stroke/systemic embolism.

**Figure 3:**
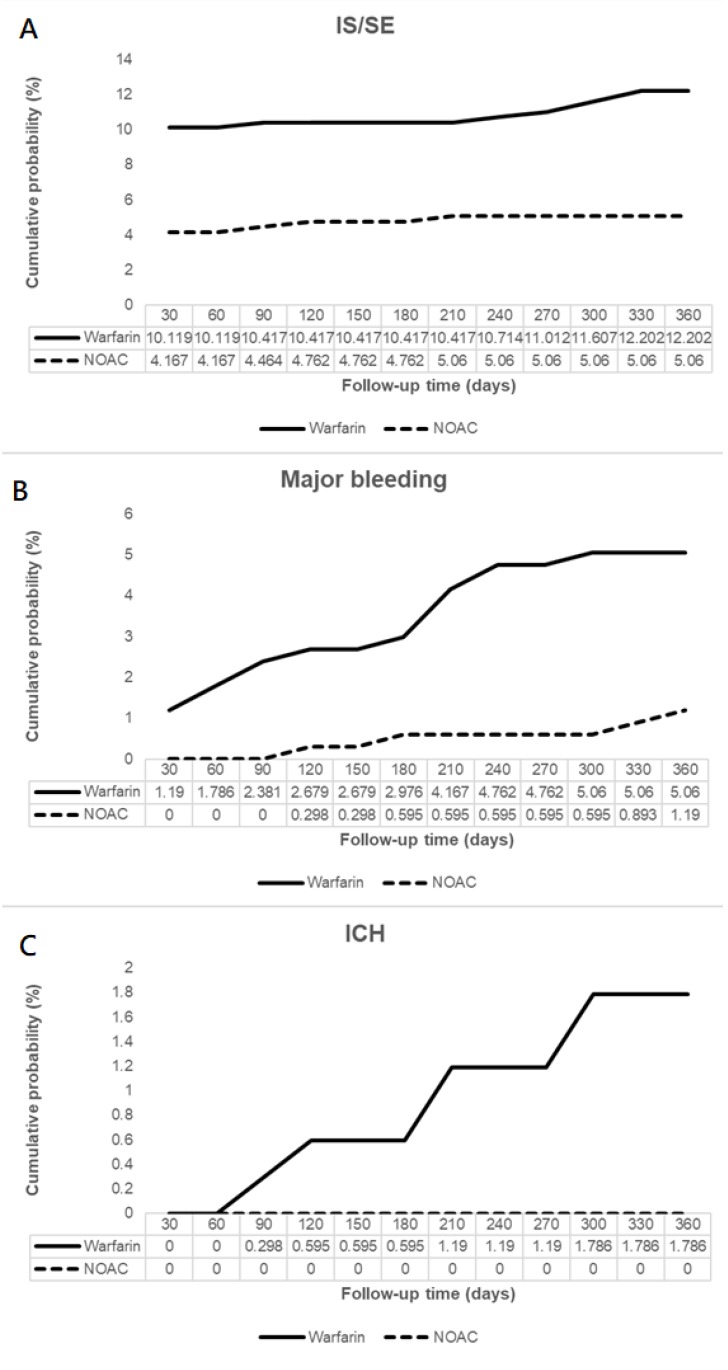
Cumulative probabilities of ischemic stroke (IS)/systemic embolism (SE) (A), major bleeding (B), and intracranial hemorrhage (ICH) (C).

Patients on NOAC had significantly reduced IS/SE (hazard ratio [HR] 0.45, 95% confidence interval [CI] 0.25-0.82), major bleeding (HR 0.21, 95% CI 0.05-0.96), and no ICH at 6 months, compared to patients on warfarin. In addition, patients on NOAC had significantly reduced IS/SE (HR 0.42, 95% CI 0.24-0.74), major bleeding (HR 0.26, 95% CI 0.09-0.76), and no ICH at 1 year, compared to patients on warfarin (**Figure 2, Figure 3**). There was no difference on GI bleeding, AMI, and death from any cause between the two groups at 6 months and at 1 year.

### Sensitivity Analysis

With enrollment criteria change to exclude patients with diagnosis of AF and cancer >1 year apart, the results were similar for IS/SE, GI bleeding, ICH, AMI, and death from any cause at 6 months of follow up, and the results were similar for IS/SE, major bleeding, ICH, AMI, and death from any cause at 1 year (**Supplementary Figure 1**). The consistent findings were the outcomes of IS/SE, ICH, AMI, and death from any cause using enrollment criteria of patients with diagnosis of AF and cancer both within 1 month and within 1 year.

## Discussions

To our knowledge, this is the first study to investigate the efficacy and safety of cancer patients with AF who were on warfarin or on NOAC in ethnic Chinese. Our study had two findings. (1) In cancer patients with AF, patients on NOAC had significant reduced IS/SE, major bleeding, and ICH compared to patients on warfarin. (2) There was no difference on GI bleeding, AMI, and death from any cause between the two groups at 6 months and at 1 year.

AF has been found to develop in patients with cancer due to comorbid diseases or direct tumor effect. Given that both diseases occurred with increased incidence in aging population, the 2 conditions are found to be coexist with increased frequency in clinical scenarios 18. Although major clinical trials of NOAC for stroke prevention in patients with AF reported benefits of reduced IS/SE and major bleeding compared use of warfarin, the trials excluded patients with cancer therefore there was the knowledge gap in the anticoagulation use in cancer patients with AF. Due to cancer related hypercoagulability, venous thromboembolism, and cancer-induced thrombocytopenia; it has been complicated to manage these patients with AF. As a matter of fact, it is possible that these patients were frequently undertreated.

A recent retrospective study compared the effectiveness of NOAC which included rivaroxaban, dabigatran, and apixaban to warfarin in patients with AF and cancer 12. The authors reported no significant difference in IS in these 3 NOACs compared to warfarin, and no significant difference in severe bleeding in rivaroxaban and dabigatran to warfarin 12. There was however significantly reduced severe bleeding in apixaban compared to warfarin (*p* = 0.01) 12. In this study however, edoxban was not included in the comparison. Current evidence of comparative treatment efficacy and bleeding complication with edoxaban and warfarin was only available from the management of venous thromboembolism in Houkosai Study 11. Although edoxaban was noninferior to subcutaneous dalteparin for recurrent venous thromboembolism, there was a significantly increased bleeding among edoxaban users 11. Another recently published study investigated provider specialty, anticoagulation, and stroke risk in patients with AF and cancer, using anticoagulation prescription filled 3 months prior to and 6 months after AF diagnosis as enrollment criteria 19. The author noted that patients with history of cancer were less likely to fill prescription with anticoagulation 19. In addition, patients with cancer were more likely to fill prescription if seen by a cardiologist with reduced risk of stroke (HR 0.89, 95% CI 0.81-0.99) and without an increased risk of bleeding (HR 1.04, 95%, CI 0.95-1.13) 19.

In a recent retrospective study, efficacy and safety of NOACs among Korean patients with AF and newly diagnosed cancer were studied. In the study which propensity score was performed for age, sex, comorbidities, CHA_2_DS_2_-VASc and HAS-BLED scores, the authors reported the significantly decreased incidence of IS/SE, major bleeding, and all-cause mortality in NOACs treated group as compared to those with warfarin. In addition, these results were not affected by different type or reduced dosage of NOACs in up to 71.9% patients in the NOACs group 20.

In this study, we retrospectively enrolled patients with AF and then screened for coexisting cancer and excluded patients with more than one type of concomitant cancer. In contrast to previous study which did not exclude patients who switched between different anticoagulants 12, our study excluded patients who had switching of OAC agents. In addition, using stricter criteria compared to previous studies 19,20, we excluded patients whose diagnoses of cancer and AF were >1 month apart, and use of OAC and AF diagnosis >1 month apart. After exclusion criteria, we then propensity score matched these patients, including age, sex, comorbidities, CHA_2_DS_2_-VASc and HAS-BLED scores, and additionally the co-administered medications, resulting 336 patients on warfarin and 336 patients on NOAC for analysis. During 6 months and 1 year follow up, we found patients on NOAC had significantly reduced IS/SE, major bleeding, and no incidence of ICH at 6 months, compared to patients on warfarin. The same results extended to 1 year with significantly reduced IS/SE, major bleeding, and still no incidence of ICH. There was no difference on GI bleeding, AMI, and death from any cause at 6 months and at 1 year. In summary, in comparison to warfarin, NOAC appears to be effective for prevention of ischemic stroke and systemic embolism in cancer patients with AF while no increased bleeding events occurred.

## Limitations

There are several limitations in epidemiologic data from NHIRD. First, using ICD-9-CM and ICD-10 codes for patient screening may miss some cases for conditions not coded correctly. Second, due to the limitation of NHIRD where detailed report of imaging study on ischemic stroke and intracranial hemorrhage were not available therefore the extent of tissue damage could not be quantified, we could still obtain incidence from the diagnosis for the occurrence of ischemia or hemorrhage. Third, we did not assess the quality of warfarin control by calculating the time in therapeutic range, since these data are not available in the NHIRD. Fourth, we did not analyze individual NOACs to delineate the efficacy and safety of each drug compared to warfarin since the number of later induced edoxaban user would be small. Further studies to generalize our study results to other populations thus are warranted.

## Conclusions

In cancer patients with AF, NOAC was associated with significant reduced ischemic stroke/systemic embolism, major bleeding, and intracranial hemorrhage compared to warfarin. NOAC may be considered in preference to warfarin in these patients.

## Supplementary Material

Supplementary figures.

## Figures and Tables

**Table 1:** Clinical characteristics of study patients

	Before matching				After matching		
Variable	On warfarin (n = 477)	On NOAC (n = 456)	P value		On warfarin (n = 336)	On NOAC (n = 336 )	P value
Characteristic							
Gender (male)	293 (61.43)	287 (62.94)	0.6338		213 (63.39)	209 (62.2)	0.7495
Mean age	73.62±10.41	75.61±9.63	0.0025		75.08±9.5	75.09±9.9	0.9910
Age group			0.0107				0.6698
18-64 years.	98 (20.55)	62 (13.6)			48 (14.29)	55 (16.37)	
65-74 years	140 (29.35)	131 (28.73)			103 (30.65)	95 (28.27)	
≥ 75 years	239 (50.1)	263 (57.68)			185 (55.06)	186 (55.36)	
Medical history							
Diabetes mellitus	163 (34.17)	194 (42.54)	0.0085		132 (39.29)	135 (40.18)	0.8130
Hypertension	338 (70.86)	369 (80.92)	0.0003		256 (76.19)	265 (78.87)	0.4055
Hyperlipidemia	158 (33.12)	225 (49.34)	<.0001		139 (41.37)	145 (43.15)	0.6394
Heart failure	134 (28.09)	129 (28.29)	0.9466		99 (29.46)	97 (28.87)	0.8652
Renal insufficiency	132 (27.67)	148 (32.46)	0.111		101 (30.06)	105 (31.25)	0.7379
Peptic ulcer disease	164 (34.38)	185 (40.57)	0.0508		129 (38.39)	133 (39.58)	0.7517
Abnormal liver function	80 (16.77)	100 (21.93)	0.0459		65 (19.35)	64 (19.05)	0.9220
Peripheral artery disease	33 (6.92)	49 (10.75)	0.039		28 (8.33)	29 (8.63)	0.8899
Prior ischemic stroke or systemic thromboembolism	85 (17.82)	74 (16.23)	0.5181		58 (17.26)	60 (17.86)	0.8393
Old myocardial infarction	24 (5.03)	27 (5.92)	0.5502		17 (5.06)	18 (5.36)	0.8622
Alcoholic history	5 (1.05)	13 (2.85)	0.0454		5 (1.49)	<5	0.7249^#^
Medication							
Antiplatelets	285 (59.75)	299 (65.57)	0.0662		214 (63.69)	226 (67.26)	0.3302
ACEi/ARB	302 (63.31)	289 (63.38)	0.9836		216 (64.29)	224 (66.67)	0.5163
Amiodarone/dronedarone	139 (29.14)	90 (19.74)	0.0008		77 (22.92)	79 (23.51)	0.855
Beta blockers	288 (60.38)	290 (63.6)	0.3114		203 (60.42)	218 (64.88)	0.2316
Calcium channel blockers	258 (54.09)	284 (62.28)	0.0112		199 (59.23)	208 (61.9)	0.4775
Diuretics	204 (42.77)	156 (34.21)	0.0073		137 (40.77)	135 (40.18)	0.8751
NSAIDs	173 (36.27)	248 (54.39)	<.0001		160 (47.62)	157 (46.73)	0.8167
Oral hypoglycemic agents	153 (32.08)	166 (36.4)	0.1636		120 (35.71)	123 (36.61)	0.8097
CHA_2_DS_2_-VASc score	3.92±1.93	4.34±1.97	0.0011		4.2±1.89	4.21±2.0	0.9369
CHA_2_DS_2_-VASc score group			0.0048				0.2946
2-3 (Moderate)	153 (32.08)	110 (24.12)			103 (30.65)	84 (25)	
4-5 (High)	157 (32.91)	176 (38.6)			114 (33.93)	129 (38.39)	
≥ 6 (Very high)	111 (23.27)	132 (28.95)			92 (27.38)	89 (26.49)	
HAS-BLED score	2.93±1.58	3.38±1.42	<.0001		3.19±1.56	3.25±1.44	0.5899
HAS-BLED score group			<.0001				0.1544
0-2 (Low)	185 (38.78)	113 (24.78)			111 (33.04)	94 (27.98)	
≥ 3 (High)	292 (61.22)	343 (75.22)			225 (66.96)	242 (72.02)	

ACEi, angiotensin converting enzyme inhibitor; ARB, angiotensin receptor blocker; COPD, chronic obstructive pulmonary disease; non-steroidal anti-inflammatory drug. #Fisher exact test Numbers <5 are not shown, as per confidentiality policies of the Taiwan National Health Insurance Research Database.
