# Supplementary material for: Novel Oral Anticoagulant versus Warfarin in Cancer Patients with Atrial Fibrillation: An 8-Year Population-Based Cohort Study
Source: J Cancer. 2020 Jan 1;11(1):92–9. doi: 10.7150/jca.36468 (PMC6930400; doi:10.7150/jca.36468)
Supplement: Supplementary file 1 — Supplementary figures. [file jcav11p0092s1.pdf]

Supplementary Figure 1
Sensitivity analysis. Primary outcomes during 6 months and 1 year follow up using exclusion criteria of patients whose cancer and AF were diagnosed >1 year apart.

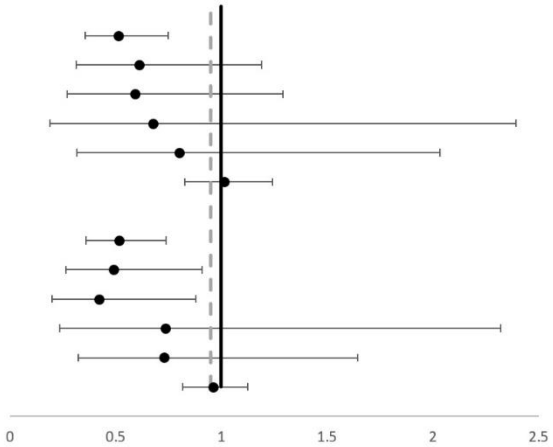

	HR	95%CI
**6 months follow-up**		
IS/SE	0.515	(0.355-0.748)
Major bleeding*	0.612	(0.315-1.19)
GIB	0.591	(0.271-1.29)
ICH	0.676	(0.191-2.395)
AMI	0.802	(0.317-2.033)
Death	1.013	(0.826-1.241)
**1 year follow-up**		
IS/SE	0.516	(0.36-0.739)
Major bleeding*	0.491	(0.265-0.91)
GIB	0.421	(0.201-0.881)
ICH	0.736	(0.234-2.321)
AMI	0.73	(0.324-1.644)
Death	0.96	(0.819-1.124)
